# Supplementary figures and images for: Transcriptional phenotype of the anti-parasitic benzodiazepine meclonazepam on the blood fluke Schistosoma mansoni
Source: PLoS Negl Trop Dis. 2025 Apr 8;19(4):e0012969. doi: 10.1371/journal.pntd.0012969 (PMC12058154; doi:10.1371/journal.pntd.0012969)

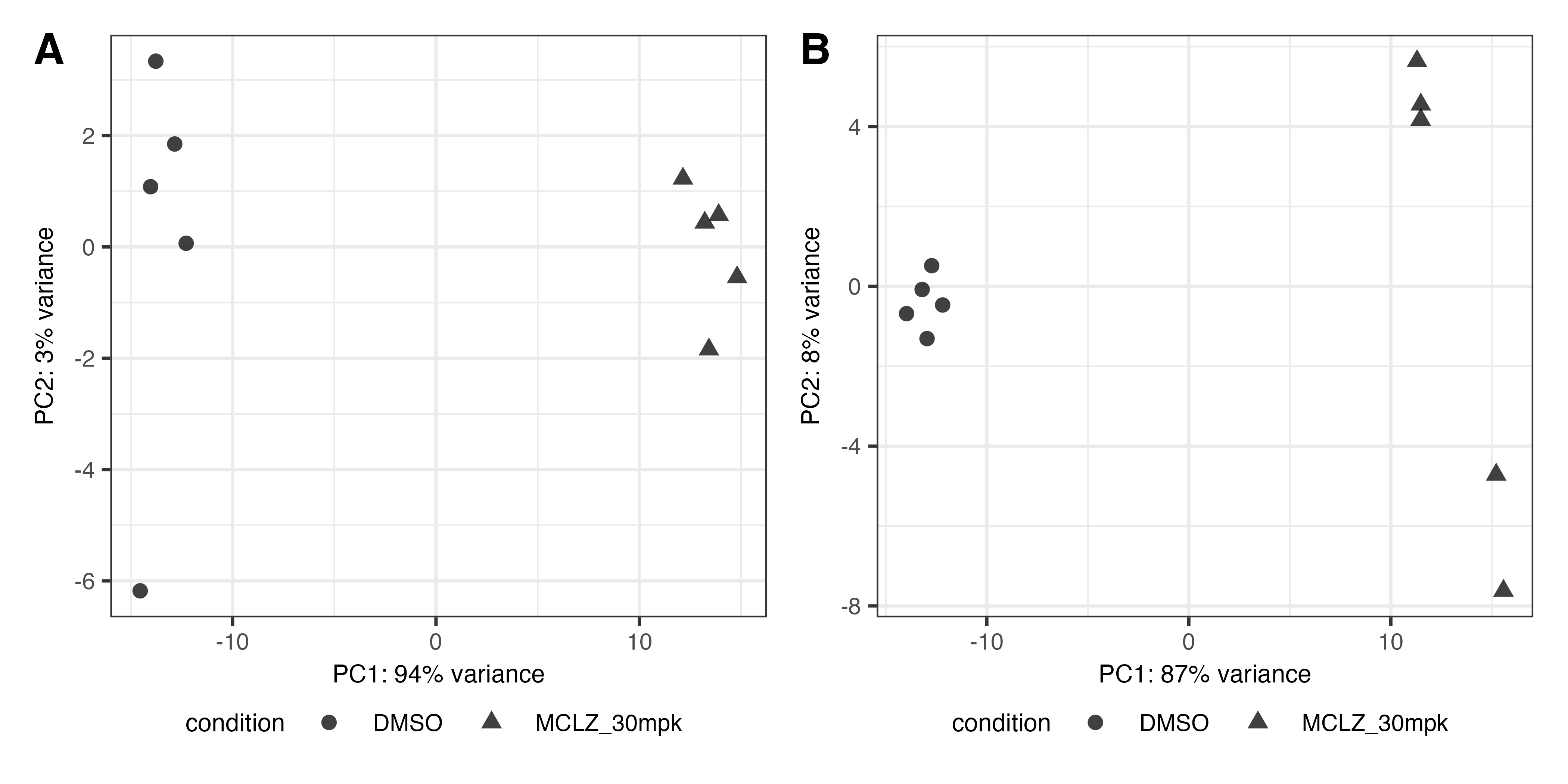

Supplement: S1 Fig — RNASeq data for four week (left) and seven week (right) infections treated with DMSO control (circles) or MCLZ (triangles). (PNG) [file pntd.0012969.s001.png]

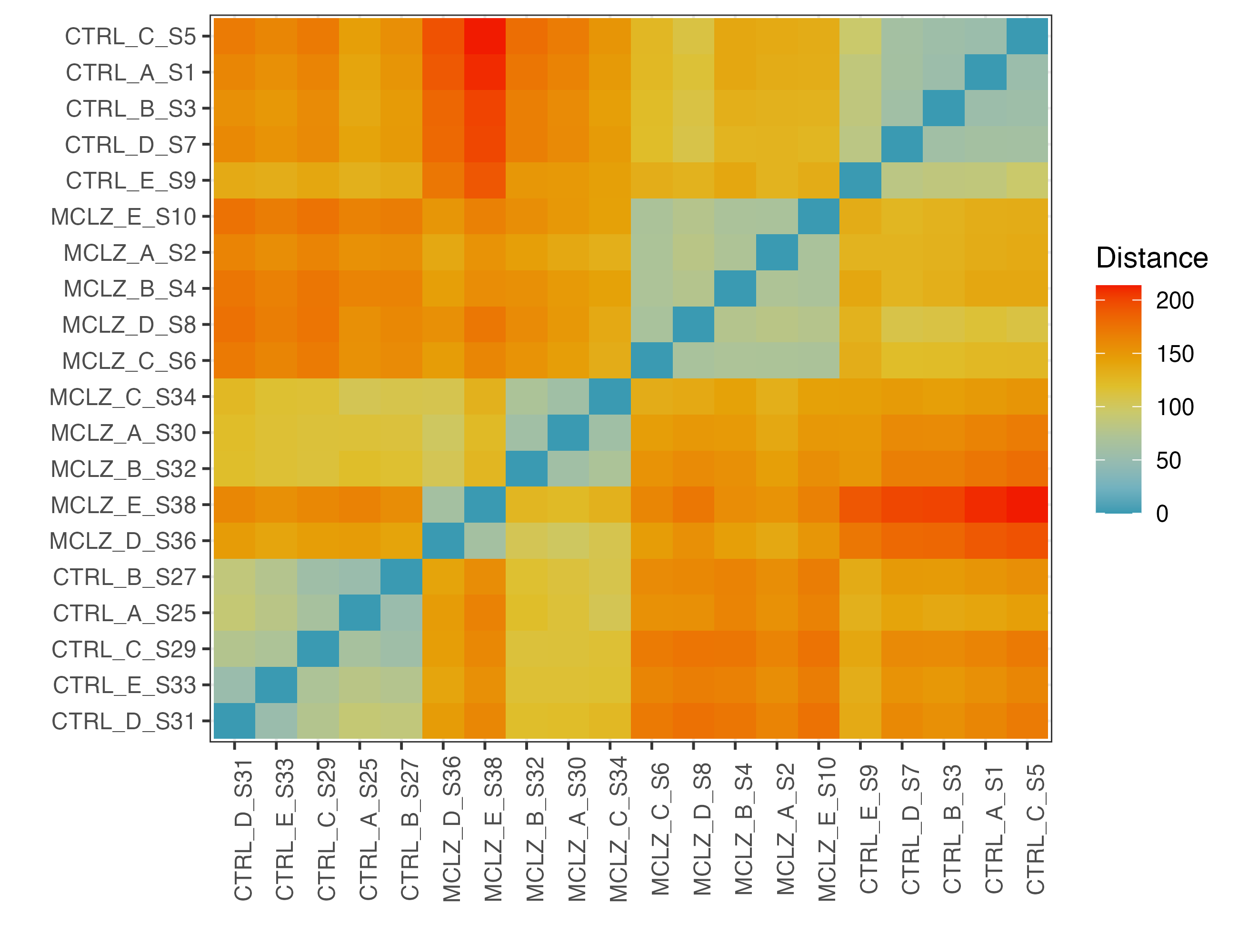

Supplement: S2 Fig — Replicates cluster based on drug treatment condition and parasite developmental stage. (PNG) [file pntd.0012969.s002.png]

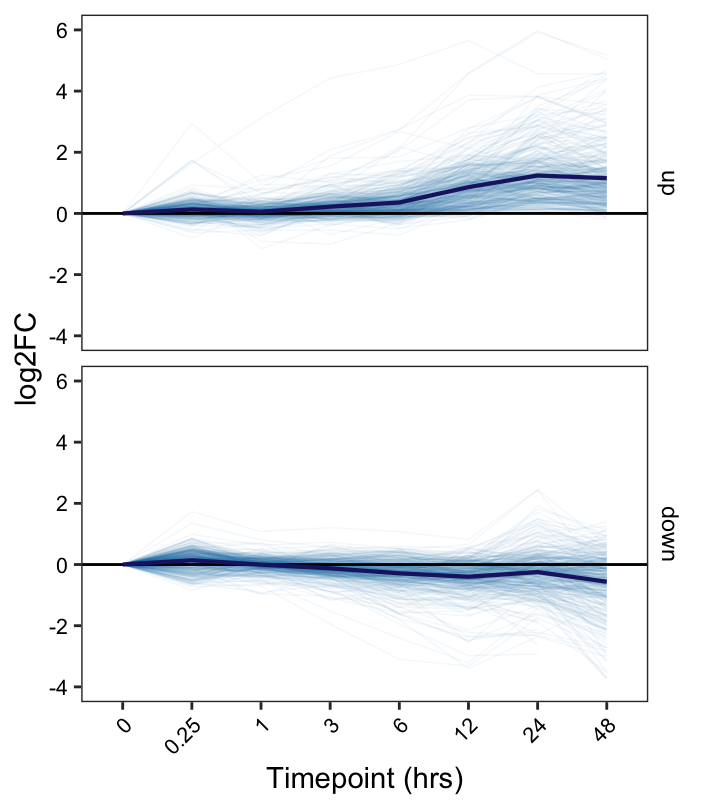

Supplement: S3 Fig — Top = expression of the 403 upregulated gene list from Fig 2B. Bottom = expression of the 400 downregulated gene list from Fig 2B. (PNG) [file pntd.0012969.s003.png]
