# Supplementary material for: Transcriptional phenotype of the anti-parasitic benzodiazepine meclonazepam on the blood fluke Schistosoma mansoni
Source: PLoS Negl Trop Dis. 2025 Apr 8;19(4):e0012969. doi: 10.1371/journal.pntd.0012969 (PMC12058154; doi:10.1371/journal.pntd.0012969)
Supplement: Attachment — Submitted filename: response_to_reviewers.pdf [file pntd.0012969.s008.pdf]

RESPONSE TO REVIEWERS

We are grateful to the reviewers and editor for the thoughtful and thorough review of our manuscript. We have revised the manuscript in response to the helpful feedback received. Our responses to each of the reviewer's specific comments are outlined below.

Methods

In vitro schistosome assays
- What was the concentration of meclonazepam and how many worms?

We have now ensured that legends for Figure 1 and Figure 4, which present data on *in vitro* schistosome assays, contain details on the drug concentrations used and the number of worms used in these assays.

- What is the drug half-life; how was 14hrs chosen as the endpoint?

While this has not been determined in a murine model, human data indicates the half-life of MCLZ is quite long (~40 hours, PMID: 3998022 in reference 83). The drug is likely metabolized faster in mice, but likely not to the extent that it is not present through this treatment window. For example, data from the initial study on meclonazepam in 1978 (PMID: 714190, reference 15) shows that mouse serum is still bioactive on worms 8 hours after dosing with MCLZ.

Generally, either 12 hour or 14 hour time points were selected for experiments based on data in Figure 1 C&D showing the onset of worm death (pro-apoptotic caspase activation). Meclonazepam was administered to mice at the end of one work day and parasites were dissected at the start of the next day. Whether an experiment was done at 12 or 14 hours was generally simply down to convenience (12 hour time points requiring an early start to the day). Data in Figure 3 show that there does not appear to be major, abrupt changes in drug response between these two time points.

Transcriptome sequencing
- "Trimmed (fastp) reads…". Missing citation?
- Missing citation for the pre-print of Schistosoma mansoni genome AND for WormBase ParaSite which should be used per WormBase ParaSite's data use policy

We appreciate the reviewers calling our attention to these oversights. Citations are now added for read trimming (reference 28), *Schistosoma mansoni* genome v10 (29) and wormbase parasite (30).

Bioinformatic analysis of divergent genes in parasitic flatworms
- Playhelminth should not be italicised

Now corrected.

Results

Reviewer #2: "…are unstudied ferritin isoforms, Smp_....". These look to be different genes entirely, not isoforms. It would be interesting to see the location of these genes in the genome and whether they are part of an operon, although perhaps out of scope for this project. I believed they are referred to as "isoforms" in at least two locations in the paper (results and discussion).

The word isoform is now removed in both instances.

Regarding TRPMs, I'm curious whether the transcripts you found to be differentially expressed are the same as those from Park et al. (https://doi.org/10.1016/j.jbc.2023.105528). They describes the activation of TRPM channel Smp_333650. I'd appreciate the author's comments on how their results either corroborate or are different from this other study.

As the reviewer notes, the study by Park et al. (reference 19) was pivotal in understanding meclonazepam action, as it identified the likely target of this drug in parasites (Smp_333650). We now specifically state whether this channel is differentially expressed in lines 294-296, which reads *"This is notable given that the putative target of MCLZ (Smp_333650, [19]) belongs to this family of ion channels, although this particular gene product was not differentially expressed."*

Our study is different from the study by Park et al. in that their focus was the identification of the precise Ca^{2+} channel MCLZ is acting on, while the focus of our work is looking at the downstream changes that occur after this event (e.g. biochemical changes in the form of pro-apoptotic caspase activation, or transcriptional changes in drug exposed parasites). Our work benefits from the prior findings from Park et al., but we address separate questions regarding the molecular mechanism of MCLZ and use different approaches. That the gene product they studied (Smp_333650) did not change in our data does not necessarily present any conflict with their results.

<u>**Conclusions**</u>

Reviewer #1: Due to the nature of the study, the results are largely descriptive. What is/are the mechanism(s) resulting in worm death? Apoptosis is induced, but how is it induced?

We have added new text elaborating on this mechanism in the first paragraph of the discussion, with reference to programmed cell death pathways that have been identified in schistosomes (lines 381-386).

<u>**Editorial and Data Presentation Modifications**</u>

Reviewer #2: Please use either "single-cell" or "single cell" throughout

We now consistently use the term "single cell."

Fig. 1B and Fig. 1D are not referenced in the main text

References to these figure panels are now included in lines 232 and 255.

Has it been determined that immune-recognition of parasites following chemotherapy is important in the MOA of meclonazepam as it is for PZQ?

This has not been determined, and we have added several sentences of new text to the discussion addressing this point (lines 467-473).

Both PZQ and MCLZ cause Ca influx in worms, through action on different Trp receptors. Would similar results be found if worms were cultured and treated in Ca-free media?

Indeed, similar results would be found between the two drugs incubated in Ca^{2+} free media. From the 1978 study (PMID: 714190, reference 15): *"When schistosomes were preincubated for 15 min in Ca^{2+} free HBS we did not observe the PZ (praziquantel) or Ro 11-3128 (meclonazepam) induced rise in the tension on the musculature of male schistosomes."*

Adding references to the sentence on page 11 'Numerous transcriptional studies …' would be helpful.

References 24-26 are now added in this sentence (line 264) for further context.

‘If, theoretically, PZQ were administered in a manner that extended _it’s_ half-life by 20 to 80 fold, it may also be effective at clearing juvenile worms.’ This is experimentally feasible: treat infected mice b.i.d. at 20, 21, and 22 days after infection.

This would be a very interesting experiment to try. However, even with bi-daily dosing PZQ is so rapidly metabolized (~0.5 hour half life; PMID: 3914644, reference 81) that the cumulative time of parasite drug exposure would likely not be equivalent to MCLZ. To address this, CYP inhibitors could be co-administered with PZQ to prevent metabolism and prolong half-life (e.g. ketoconazole as in PMID:18021336 or 1-aminobenzotriazole as in PMID: 28934207). We raised the prospect of this experiment in the discussion because it is intriguing, but beyond the scope of the current study.

Journal Requirements

All figures are now provided in .tif format and have been processed using the PACE digital diagnostic tool. An ‘Author Summary’ written for a lay audience is now provided after the introduction.
